# Optimized Enzymatic Synthesis of Feruloyl Derivatives Catalyzed by Three Novel Feruloyl Esterases from *Talaromyces wortmannii* in Detergentless Microemulsions

**DOI:** 10.1016/j.csbj.2018.09.005

**Published:** 2018-10-05

**Authors:** Io Antonopoulou, Laura Iancu, Peter Jütten, Alexander Piechot, Ulrika Rova, Paul Christakopoulos

**Affiliations:** a Biochemical Process Engineering, Division of Chemical Engineering, Department of Civil, Environmental and Natural Resources Engineering, Luleå University of Technology, Luleå SE-97187, Sweden; b Dupont Industrial Biosciences, Nieuwe Kanaal 7-S, Wageningen 6709, the Netherlands; c Taros Chemicals GmbH & Co KG, Emil-Figge-Str. 76a, Dortmund 44227, Germany

## Abstract

Three novel feruloyl esterases (Fae125, Fae7262 and Fae68) from *Talaromyces wortmannii* overexpressed in the C1 platform were evaluated for the transesterification of vinyl ferulate with two acceptors of different size and lipophilicity (prenol and L-arabinose) in detergentless microemulsions. The effect of reaction conditions such as the microemulsion composition, the substrate concentration, the enzyme load, the pH, the temperature and the agitation were investigated. The type A Fae125 belonging to the subfamily 5 (SF5) of phylogenetic classification showed highest yields for the synthesis of both products after optimization of reaction conditions: 81.8% for prenyl ferulate and 33.0% for L-arabinose ferulate. After optimization, an 8-fold increase in the yield and a 12-fold increase in selectivity were achieved for the synthesis of prenyl ferulate.

## Highlights

• Three feruloyl esterases from *Talaromyces wortmannii* were tested for their synthetic and hydrolytic activity.
• Reaction conditions were optimized for the synthesis two feruloyl derivatives, prenyl ferulate and L-arabinose ferulate.
• Fae125 offered highest yield for both products (81.8% and 33%, respectively).

## Graphical Abstract

**Figure:**
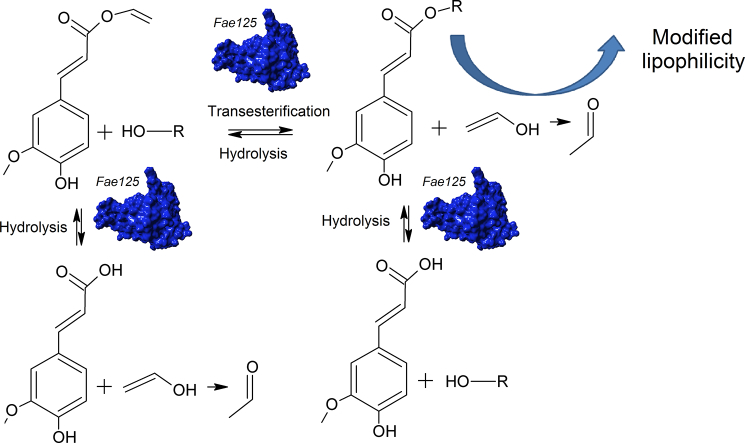

## 1 Introduction

Feruloyl esterases (FAEs, EC 3.1.1.73) represent a subclass of carboxyl esterases (EC 3.1.1.1) belonging to the 1CE family of the CAZy database (www.cazy.org). Under normal conditions, they catalyze the hydrolysis of the ester bond between hydroxycinnamic acids such as ferulic acid (FA) and sugars such as L-arabinose and D-galactose during enzymatic degradation of lignocellulosic biomass. FAEs have initially been categorized into four types (A-D) based on their specificity towards mono-ferulate and di-ferulates, for methoxy- or hydroxy- substitutions on the phenolic ring and on their amino-acid sequence identity [1]. Later, a phylogenetic classification was proposed categorizing this diverse class of enzymes into thirteen subfamilies (SF1–13) [2,3].

Except for their role as synergistic hydrolytic enzymes, FAEs have been utilized as biosynthetic tools for the derivatization of hydroxycinnamates via (trans) esterification reactions resulting in products with modified lipophilicity and often improved bioactive properties such as antioxidant activity [4]. Since the first application of a FAE for the esterification of FA with 1-pentanol [5], research has been focused on the modification of ferulates with alcohols or sugars in ternary systems forming detergentless microemulsions or solvent-free systems catalyzed by free and immobilized commercial enzymatic preparations and FAEs derived from fungi such as *Aspergillus niger*, *Talaromyces stipitatus* and *Myceliophthora thermophila* [[6], [7], [8], [9], [10], [11], [12], [13], [14], [15]]. The advantages of enzymatic transesterification include mild operating conditions, high selectivity, one-step synthesis and easier downstream processing while they can contribute to the development of more sustainable processes for manufacturing bioactive components for the food, cosmetics and pharmaceutical industry [16].

**Fig. 1:**
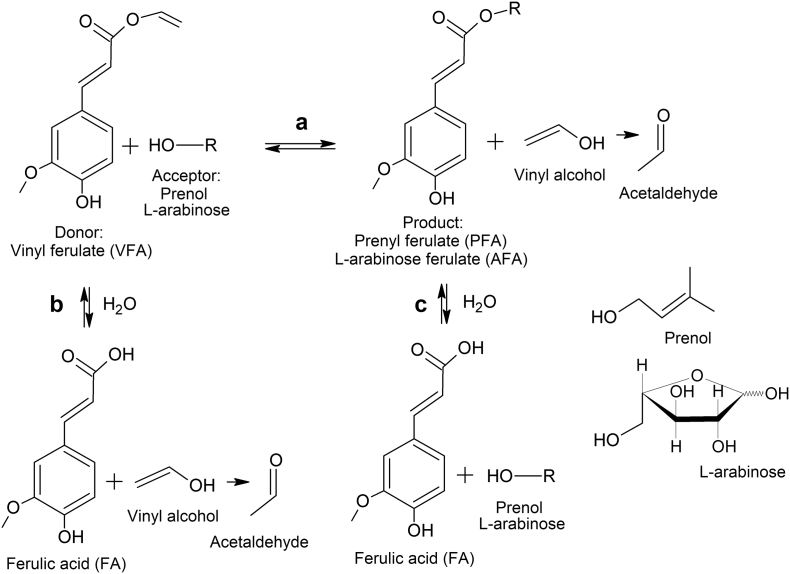
Schematic representation of enzymatic a) transesterification of VFA (donor) with prenol or L-arabinose (acceptor) b) hydrolysis of VFA (competitive side-reaction) c) hydrolysis of product (competitive side-reaction). Under normal conditions vinyl alcohol tautomerizes to acetaldehyde.

The objective of this work is the evaluation of three novel FAEs (Fae125, Fae7262 and Fae68) from *Talaromyces wortmannii* as for their ability to synthesize two targeted products of different lipophilicity namely, prenyl ferulate (PFA) and L-arabinose ferulate (AFA). The synthesis of the aforementioned compounds was done by the transesterification of the activated donor, vinyl ferulate (VFA), with the respective acceptor (prenol or L-arabinose) monitoring the competitive side hydrolytic reaction(s) (Fig. 1). The reaction conditions such as the system composition, the substrate concentration, the enzyme load, the pH and the temperature were optimized aiming to the development of competitive transesterification reactions offering increased yield and selectivity.

## 2 Materials and Methods

### 2.1 Enzymes and Materials

The feruloyl esterases Fae125, Fae7262 and Fae68 from *T. wortmannii*, over-expressed individually in low background C1-expression strains, were provided by DuPont Industrial Biosciences (Wageningen, the Netherlands) in the form of lyophilized powder. Methyl ferulate (MFA) was purchased from Alfa Aesar (Karlsruhe, Germany). *n*-Hexane (<0.02% water), *t*-butanol (anhydrous, ≥ 99.5%), prenol (99%), L-arabinose (≥99%), FA, MOPS solution 1 M and other chemicals were purchased from Sigma-Aldrich (Saint Louis, USA). VFA was provided by Taros Chemicals GmbH & Co. KG (Dortmund, Germany).

### 2.2 Protein and Enzyme Assays

Stock solutions of enzymes were prepared by dilution in 100 mM MOPS-NaOH pH 6.0. The protein concentration of the enzyme solutions was determined by the Pierce BCA Protein Assay (Thermofisher Scientific, Waltham, USA).. The enzyme hydrolytic activity was assayed in 100 mM MOPS-NaOH pH 6.0 at 1 mM substrate (20 μL of 50 mM stock solution prepared in dimethyl sulfoxide) at 45 °C for 10 min using varying enzyme load (0.005–5 μg FAE mL^−1^) at a total volume of 1 mL. The reactions were ended by incubation at 100 °C for 10 min and subsequent dilution in acetonitrile. The PFA synthetic activity was assayed in 53.4: 43.4: 3.2 *v*/v/v *n*-hexane: *t*-butanol: 100 mM MOPS-NaOH pH 6.0 at 5 mM VFA and 200 mM prenol while the AFA synthetic activity was assayed at 19.8: 74.7: 5.5 v/v/v *n*-hexane: *t*-butanol: 100 mM MOPS-NaOH pH 6.0 at 5 mM VFA and 55 mM L-arabinose at varying enzyme load (0.005–0.02 mg FAE mL^−1^) at 45 °C for 10 min. The reactions were ended by dilution in acetonitrile. All reactions were performed in duplicate accompanied by appropriate blank samples, containing buffer instead of enzyme. One unit (1 U) is defined as the amount of enzyme (mg) releasing 1 μmol product per minute under the defined conditions.

### 2.3 Transesterification Reactions

Transesterification of VFA with prenol or L-arabinose was carried out in a ternary system forming detergentless microemulsions (*n*-hexane: *t*-butanol: buffer) at a total volume of 500 μL. The reaction mixtures were prepared as following: amount of VFA donor was diluted in a mixture of *n*-hexane and *t*-butanol followed by vigorous shaking. In the case of PFA synthesis, prenol was added prior to addition of enzyme in the form of concentrated stock solution in buffer and followed by vigorous shaking. In the case of AFA synthesis, L-arabinose and enzyme were introduced in the form of stock solution in buffer followed by vigorous shaking. The effect of medium composition was studied in four different systems (*n*-hexane: *t*-butanol: buffer): system I (37.8:57.2: 5.0 *v*/v/v), system II (53.4: 43.4: 3.2 v/v/v), system III (47.2: 50.8: 2.0 v/v/v) and system IV (19.8: 74.7: 5.5 v/v/v). The effect of donor concentration was studied in a range of 20–150 mM VFA while the effect of acceptor concentration was studied in a range of 0.1–3 M prenol or 10–80 mM L-arabinose. The effect of pH was studied using the following buffers (100 mM): sodium acetate (4–6), MOPS-NaOH (6–8), Tris-HCl (8–10). The effect of temperature was studied in a range of 25-60 °C. In each study, optimal conditions offering the highest PFA or AFA concentration in the mixture were applied in subsequent experiments. Unless otherwise stated, reactions were carried out in a temperature-controlled water bath at 50 mM VFA, 200 mM prenol or 30 mM L-arabinose, 40 °C, 100 mM MOPS-NaOH pH 6.0, 0.004 mg FAE mL^−1^ Fae125, Fae7262 and 0.01 mg FAE mL^−1^ Fae68 for 8 h of incubation without agitation. Experiments including agitation (1000 rpm) were performed in an Eppendorf Thermomixer (Eppendorf, Hamburg, Germany). All reactions were ended by addition of acetonitrile and were performed in duplicate accompanied by appropriate blank samples, containing buffer instead of enzyme.

### 2.4 Quantitative Analysis of Feruloyl Compounds

Analysis of ferulates (donor and products) was performed by HPLC using a 100–5 C18 Nucleosil column (250 mm × 4.6 xx) (Macherey Nagel, Düren, Germany) at isocratic conditions (70:30 *v*/v acetonitrile: water), 0.6 mL min^−1^ and room temperature while detection was done by a PerkinElmer Flexar UV/Vis detector at 300 nm as described previously [11,12]. The yield was defined as the molar amount of generated transesterification product compared to the initial amount of limiting reactant, expressed as percentage. The overall conversion of VFA was defined as the sum of molar amount of products (PFA or AFA and FA) compared to the initial amount of donor, expressed as percentage. The rate was expressed as the molar concentration of generated transesterification product per hour per amount of enzyme. The selectivity was defined as the molar concentration of generated transesterification product divided by the concentration of generated FA.

### 2.5 Bioinformatic Tools

The sequences of Fae125 (Genbank ID: MF362595) and FaeA1 from *M. thermophila* C1 (Genbank ID: JF826027) were aligned by CLUSTALW [17,18]. The secondary structure prediction of Fae125 was done by homology modeling using the embedded tool in YASARA Structure [19]. Five PDB entries from Uniref90 (EMBL-EBI) were identified as templates for homology modeling. Twenty-one models were built based on the templates and the final exported model was a hybrid model based on the best part of each model, resulting in higher quality scores (*Z*-score). Based on YASARA, the quality of model is bad when Z-score values range between −4 and − 3: bad and optimal when values are at a range between 0 and 4.

## 3 Results and Discussion

### 3.1 Hydrolytic and Synthetic Activity

The FAE hydrolytic activity towards various hydroxycinnamates (PFA, VFA, MFA, AFA) and the FAE synthetic activity towards the two targeted compounds PFA and AFA was tested at fixed conditions (45 °C, 10 min of incubation) (Table 1). Fae68 showed the highest hydrolytic activity for all substrates with >10-fold activity for lipophilic substrates compared to Fae125 and Fae7262. All tested FAEs showed increased hydrolytic activity towards AFA compared to small substituted FA derivatives such as VFA and MFA, revealing that these enzymes have increased specificity towards natural substrates comparing to synthetic ones. This trend is supported by previous reports on the characterization of FAEs based on hydrolysis of natural and synthetic substrates [6,[17], [18], [19]]. Synthesis at fixed conditions showed that only Fae125 was able to synthesize PFA and AFA while Fae7262 showed synthetic activity only towards PFA. Fae68 showed no synthetic activity at given conditions. It was observed that there is no direct correlation between the synthetic and hydrolytic activity of tested FAEs, since Fae68 had very high hydrolytic activity but no synthetic activity during this preliminary experiment. The reaction conditions for synthesis of the two targeted compounds, PFA and AFA, were optimized in following experiments using the three FAEs from *T. wortmannii* as biocatalysts.

**Table 1:** Biochemical characteristics of FAEs from *T. wortmannii.*

Enzyme	Genbank ID	Type	Subfamily^a^	FAE content^b^ (mg FAE mg^−1^ protein)	Calculated MW^c^ (kDa)	Specific activity (U mg^−1^ FAE)					
						Hydrolysis				Synthesis	
						PFA	VFA	MFA	AFA	PFA	AFA
Fae125	MF362595.1	A	SF5	0.10	33.9	14.0 ± 3.9	13.4 ± 1.0	10.3 ± 2.0	**17.3** ± 0.2	0.713 ± 0.054	0.244 ± 0.036
Fae7262	MF362597.1	B	SF6	0.15–0.25	35.8	16.3 ± 2.9	16.1 ± 1.1	13.6 ± 0.5	**264.3** ± 42.5	0.659 ± 0.146	n/d
Fae68	MF362596.1	B	SF1	0.10–0.15	58.7	199.0 ± 65.8	148.5 ± 19.3	176.1 ± 3.3	**387.6** ± 10.7	n/d	n/d

a Phylogenetic classification according to Dilokpimol et al. [3].

b Αccording to Antonopoulou et al. [16].

c Calculated by the ExPASy-ProtParam tool; n/d: not detected; the errors represent the standard error from regression.

### 3.2 Effect of System Composition

**Fig. 2:**
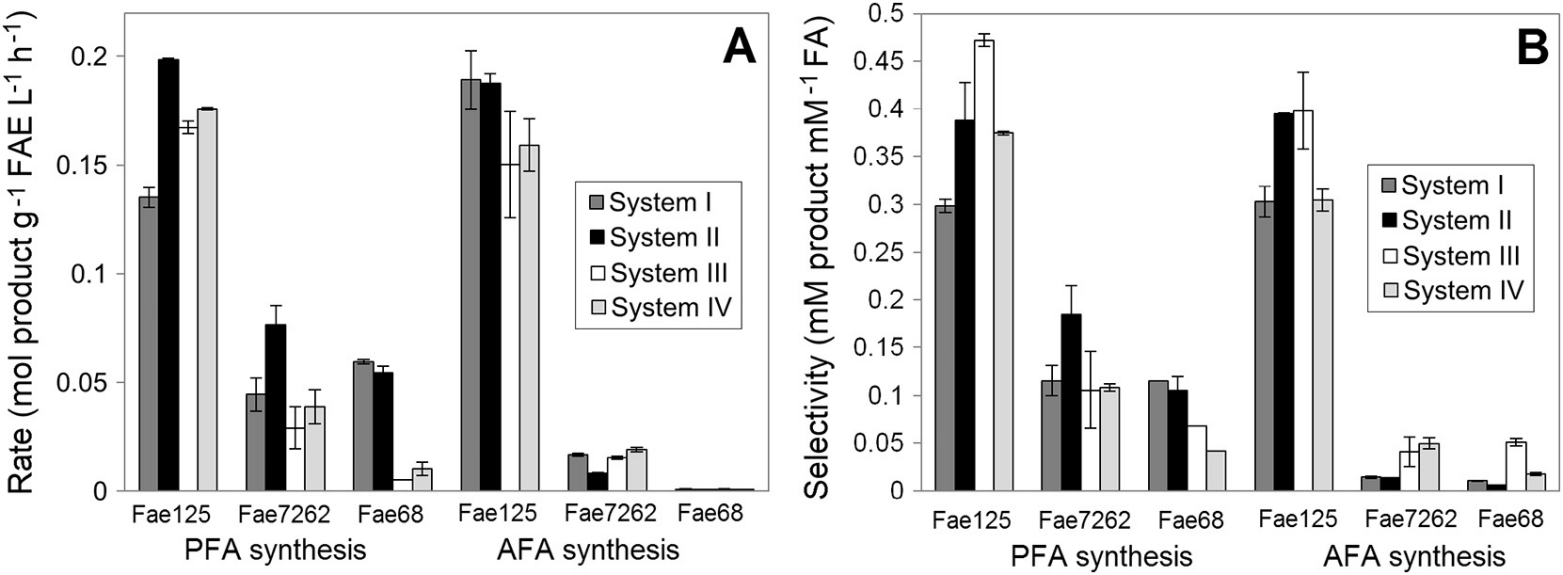
Effect of medium composition on the a) rate and b) selectivity. Reactions were carried out in *n*-hexane: *t*-butanol: 100 mM MOPS-NaOH pH 6.0, 50 mM VFA, 200 mM prenol or 30 mM L-arabinose, 40 °C, 8 h. Dark gray: system I (37.8: 57.2: 5.0 v/v/v), black: system II (53.4: 43.4:3.2 v/*v*/v), white: system III (47.2: 50.8: 2.0 *v*/v/v), light gray: system IV (19.8: 74.7: 5.5 v/v/v).

Transesterification was carried out at four different compositions of *n*-hexane: *t*-butanol: 100 mM MOPS-NaOH pH 6.0 at fixed conditions (50 mM VFA, 200 mM prenol or 30 mM L-arabinose, 8 h, 40 °C). Highest rate, yield and selectivity were observed when Fae125 was used as biocatalyst during the synthesis of both products (PFA and AFA) followed by Fae7262 and Fae68. Selectivity was <1 for both products in all tested systems showing that hydrolysis is the predominant reaction over transesterification. Regarding PFA synthesis, Fae125 showed improved rate (0.198 mol PFA g^−1^ FAE L^−1^ h^−1^) at system II (53.4: 43.4: 3.2 *v*/v/v) (Fig. 2A) and the highest selectivity (0.471) was observed at lowest water content (system III; 47.2: 50.8: 2.0 v/v/v) (Fig. 2B). Fae7262 showed improved rate and selectivity (0.077 mol PFA g^−1^ FAE L^−1^ h^−1^ and 0.185) at system II while Fae68 at system I (37.8: 57.2: 5.0 v/v/v). The system offering highest product concentration was considered as optimal for subsequent experiments in PFA synthesis: system II for Fae125, Fae7262 and system I for Fae68. Fae125 showed similar rates with PFA synthesis when L-arabinose was used as acceptor, exhibiting the highest rates (0.189 mol AFA g^−1^ FAE L^−1^ h^−^1) at system I or system II and the highest selectivity, equal to 0.663, at lower water content (system II or system III). System I was selected as optimal, as the increased water content would allow the possibility of adding higher acceptor concentration in soluble form during subsequent experiments. AFA synthesis was negligible (<2%) for Fae7262 and Fae68. The following systems offering highest product concentration were considered as optimal for subsequent AFA synthetic experiments: system I for Fae125 and Fae68 and system IV for Fae7262.

### 3.3 Effect of Donor Concentration

**Fig. 3:**
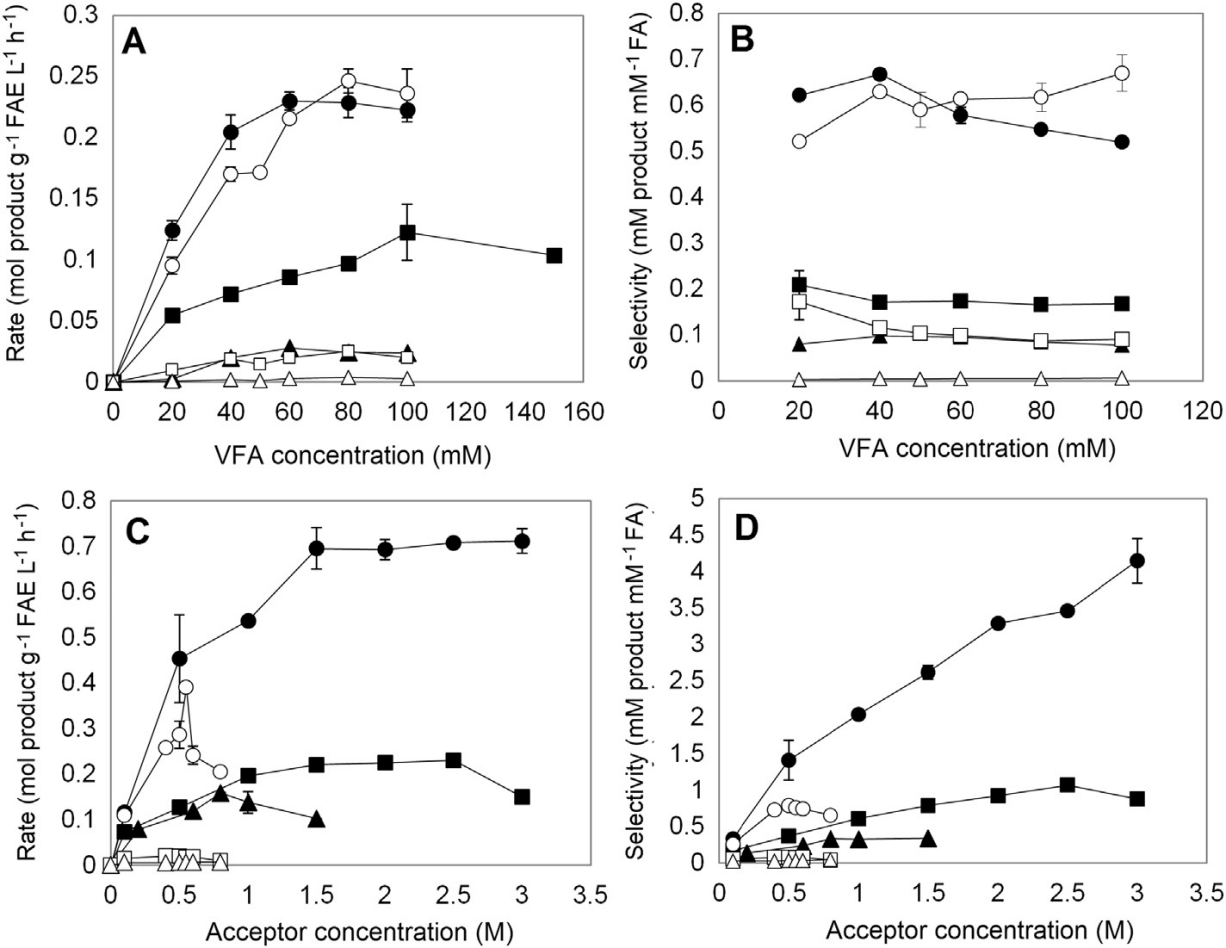
Effect of substrate concentration on the A, C) rate and B, D) selectivity. Reactions were carried out at optimal system for each enzyme, 100 mM MOPS-NaOH pH 6.0, 40 °C for 8 h. The effect of donor concentration was determined at 200 mM prenol or 30 mM L-arabinose. The effect of acceptor concentration was determined at optimal donor concentration for each enzyme. Black marker: PFA; White marker: AFA. Circle: Fae125, Square: Fae7262, Triangle: Fae68.

The effect of donor concentration on the rate and selectivity was studied under optimal medium composition for each enzyme. During PFA synthesis, Fae125 exhibited the highest rate at 60 mM VFA (0.230 mol PFA g^−1^ FAE L^−1^ h^−1^) with a selectivity of 0.579 (Fig. 3A). A 2-fold increase in VFA concentration (from 50 mM to 100 mM) resulted in a 30% decrease in selectivity for Fae125 (Fig. 3B). Highest rate was achieved at 100 mM by Fae7262 (0.122 mol PFA g^−1^ FAE L^−1^ h^−1^) and at 60 mM by Fae68 (0.028 mol PFA g^−1^ FAE L^−1^ h^−1^). Selectivity was not affected by varying the VFA concentration for Fae7262 and Fae68 (0.177 and 0.087, respectively). During AFA synthesis, reactions catalyzed by Fae125 resulted in similar rates with PFA synthesis and in particular the highest rate was observed at 80 mM VFA (0.246 mol AFA g^−1^ FAE L^−1^ h^−1^). At 80 mM VFA, Fae125 converted 42.9% of donor into products while AFA yield was 26.2%. By increasing the VFA concentration, selectivity remained constant at 0.596. When Fae7262 and Fae68 were used, rates were significantly lower and achieved yields were negligible (<2%) showing optimum at 80 mM VFA. In all cases, hydrolysis was a predominant reaction.

### 3.4 Effect of Acceptor Concentration

The effect of acceptor concentration (prenol or L-arabinose) was examined at optimal medium composition and donor concentration for each enzyme. When prenol was used as acceptor, highest rate was observed at 1.5 M for Fae125 and Fae7262 and at 0.8 M for Fae68 (Fig. 3C). In more details, Fae125 showed the highest rate (0.696 mol PFA g^−1^ FAE L^−1^ h^−1^) and yield (37.1%) at increased prenol concentration that remained constant at the range 1.5–3.0 M of prenol. Fae125 selectivity had a linear increase by increasing the prenol concentration reaching a value of 4.148 at 3 M acceptor (Fig. 3D). Fae7262 showed the highest rate (0.220 mol PFA g^−1^ FAE L^−1^ h^−1^) at a range of 1.5–2.5 M prenol which was decreased by 32% at 3 M prenol. Fae68 was the least stable enzyme showing a linear decrease in rate at prenol concentrations higher than 0.8 M while its selectivity remained constant between 0.8 and 1.5 M and equal to 0.342.

When L-arabinose was used as acceptor, highest rates were achieved at 0.50 M L-arabinose for all tested enzymes while the rate was decreased or stabilized at concentrations >0.50 M. This trend does not reflect possible limitations on enzyme specificity but process limitations imposed by the insolubility of L-arabinose in organic solvents creating the need of introducing this acceptor in a solubilized form only as part of the water phase in the form of stock solution. In this case and at 5.0% water phase (system I), a stock solution of 1 M L-arabinose would be prepared that being on its solubility limit at 25 °C. The process limitations imposed by the insolubility of sugars are reflected in achieved rates for Fae125, an enzyme that appears to catalyze the transesterification of VFA with prenol and L-arabinose in similar rates up to this stage. As shown in Fig. 3C, both PFA and AFA rates are at similar up to 0.5 M acceptor (0.454 mol PFA g^−1^ FAE L^−1^ h^−1^ and 0.390 mol AFA g^−1^ FAE L^−1^ h^−1^, respectively). However, at concentrations >0.5 M the transesterification rate for AFA synthesis is decreased by 50% while the rate for PFA synthesis is increased dramatically offering significantly higher selectivity and yields, as prenol is soluble in the organic solvent mixture at high concentrations.

### 3.5 Kinetics

The apparent kinetic constants for each substrate were determined by non-linear regression (p < 0.0001) fitting the Michaelis-Menten equation on acquired data presentedin previous paragraphs regarding the effect of substrate concentration on the transesterification rate. Generally, Fae125 exhibited the highest rate (*V*_*max*_), turnover rate (*k*_*cat*_) and catalytic efficiency (*k*_*cat*_/*K*_*m*_) followed by Fae7262 and Fae68 for both products (PFA and AFA synthesis) (Table 2). Regarding PFA synthesis, the FAEs belonging to phylogenetic subfamilies related to acetyl xylan esterases, such as Fae125 (SF5) and Fae7262 (SF6) showed similar specificity (*K*_*m*_) for both substrates (~20 mM). On the contrary, the tannase related Fae68 belonging to SF1 showed the highest specificity (lowest *K*_*m*_) for both substrates (VFA and prenol) although the turnover rate was 15-fold and 4-fold lower compared to Fae125 for VFA and prenol, respectively. Regarding AFA synthesis, Fae125 and Fae7262 showed >2-fold higher specificity (~50 mM) towards VFA compared to Fae68. Fae7262 and Fae68 exhibited considerably low catalytic efficiency, since AFA synthesis was <2% for both enzymes. In comparison with our previous work [11,12], Fae125 shows similar rate, specificity and catalytic efficiency with SF6 FAEs from *M. thermophila* C1 such as FaeB1 and FaeB2 rather than the SF5 FAEs such as FaeA1 and FaeA2 for both products. It is important to highlight that Fae125 has similar turnover rate for VFA independently of the acceptor, while the reduced turnover rate for L-arabinose comparing to prenol reflects the process limitations derived from the low solubility of L-arabinose in the reaction system.

**Table 2:** Apparent Michaelis-Menten kinetic constants.

Enzyme	VFA				Prenol			
	*V*_*ma*x_ (mol PFA g^−1^ FAE L^−1^ h^−1^)	*K*_*m*_ (mM)	*k*_*cat*_ (10^3^ g^−1^ FAE min^−1^)	*k*_*cat*_/*K*_*m*_ (mM^−1^ g^−1^ FAE L^−1^ min^−1^)	*V*_*max*_ (mol PFA g^−1^ FAE L^−1^ h^−1^)	*K*_*m*_ (mM)	*k*_*cat*_ (10^3^ g^−1^ FAE min^−1^)	*k*_*cat*_/*K*_*m*_ (mM^−1^ g^−1^ FAE L^−1^ min^−1^)
Fae125	0.290 ± 0.029	21.2 ± 7.4	40.9 ± 4.0	1932 ± 703	0.854 ± 0.039	487 ± 87	120.6 ± 5.5	248 ± 45
Fae7262	0.124 ± 0.004	26.2 ± 2.9	23.1 ± 0.8	880 ± 101	0.272 ± 0.088	416 ± 111	50.6 ± 3.5	122 ± 34
Fae68	0.0283 ± 0.001	16.3 ± 3.7	2.768 ± 0.127	170 ± 40	0.190 ± 0.030	287 ± 148	18.6 ± 3.0	64.9 ± 35.2


Enzyme	VFA				L-arabinose			
	*V*_*max*_ (mol AFA g^−1^ FAE L^−1^ h^−1^)	*K*_*m*_ (mM)	*k*_*cat*_ (10^3^ g^−1^ FAE L^−1^ min^−1^)	*k*_*cat*_/*K*_*m*_ (mM^−1^ g^−1^ FAE L^−1^ min^−1^)	*V*_*max*_ (mol AFA g^−1^ FAE L^−1^ h^−1^)	*K*_*m*_ (mM)	*k*_*cat*_ (10^3^ g^−1^ FAE L^−1^ min^−1^)	*k*_*cat*_/*K*_*m*_ (mM^−1^ g^−1^ FAE L^−1^ min^−1^)
Fae125	0.382 ± 0.049	51.3 ± 14.8	54.0 ± 6.9	1053 ± 332	0.369 ± 0.070	20.6 ± 11.7	52.1 ± 9.8	2521 ± 1503
Fae7262	0.025 ± 0.005	55.5 ± 15.3	7.645 ± 0.970	138 ± 42	0.0255 ± 0.004	18.1 ± 10.3	4.8 ± 0.8	262.3 ± 153.6
Fae68	0.009 ± 0.003	130.4 ± 67.7	0.871 ± 0.264	6.7 ± 4.0	0.009 ± 0.001	20.5 ± 10.5	0.8 ± 0.1	40.6 ± 21.5

The errors represent the standard error from regression.

### 3.6 Effect of Enzyme Concentration

**Fig. 4:**
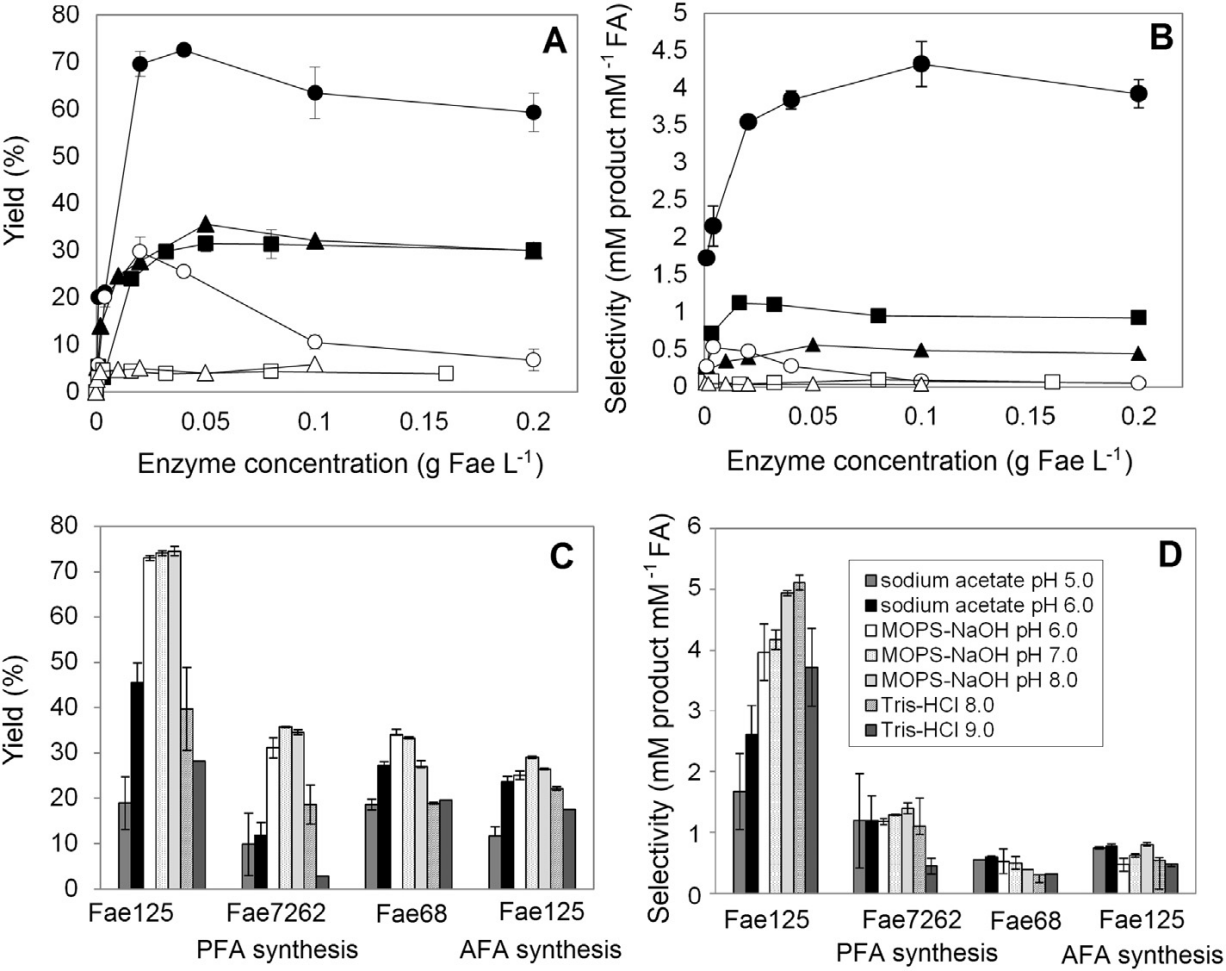
Effect of enzyme concentration on the A) yield and B) selectivity. Reactions were carried out at 100 mM MOPS-NaOH pH 6.0, 40 °C and optimal system and substrate concentration of each enzyme for 8 h. Black marker: PFA; White marker: AFA. Circle: Fae125, Square: Fae7262, Triangle: Fae68. Effect of pH on the C) yield and D) selectivity. Reactions were carried out at 40 °C and optimal system, substrate and enzyme concentration of each enzyme for 8 h.

In bioconversion applications, aim is usually the reduction of enzyme load without compromising the yield as the high production cost of enzymes is the main limiting factor for industrial application. In our study, the effect of enzyme concentration was studied at optimal medium composition and substrate concentration of each tested enzyme. During PFA synthesis, highest yield was observed at 0.04 g FAE L^−1^ for Fae125 and 0.05 g FAE L^−1^ for Fae7262 and Fae68 (72.0, 31.3 and 35.3% yield, respectively) (Fig. 4A). Highest selectivity was observed at 0.1, 0.003 and 0.05 g FAE L^−1^ for Fae125, Fae7262 and Fae68, respectively (Fig. 4B). Thus, the observed reduction in the yield over 0.04 g FAE L^−1^ for Fae125 indicates reduced activity at very high enzyme loads as it appears that a dynamic equilibrium has been achieved between hydrolytic and synthetic reactions (Fig. 1). During AFA synthesis, the highest yield was observed in the case of Fae125 (29.7%) at halved enzyme load (0.02 g FAE L^−1^) while over this value the yield was dramatically decreased (up to 80% decrease). In the same time, the selectivity was dramatically decreased over 0.004 g FAE L^−1^, revealing that after a critical ratio between AFA and VFA is reached the enzyme tends to hydrolyze AFA (reaction c in Fig. 1). At high enzyme loads this phenomenon is more evident and it could be assessed at 8 h of incubation. Last, use of Fae7262 and Fae68 resulted in very low yields (<5%) at varying enzyme concentration (0–0.2 g FAE mL^−1^).

### 3.7 Effect of pH

A variation in pH may affect the ionization state of the residues at the active site of the enzyme influencing activity and more specifically transesterification efficiency. We examined the effect of pH (range 5–9) on the yield and selectivity at optimal medium composition, substrate and enzyme concentration for each enzyme. Increased yield, rate and selectivity were observed at a range of pH 6–8 using 100 mM MOPS-NaOH for each enzyme while buffers sodium acetate and Tris-HCl appeared to have a negative impact on the enzyme stability. More specifically, highest PFA yield and rate was observed for Fae125 (73–74.5% and 0.456–0.232 mol PFA g^−1^ FAE L^−1^ h^−1^) at a pH range (6–8) when MOPS-NaOH was used as buffer (Fig. 4C). Selectivity was 20% higher at pH 8.0 compared to pH 6.0 (Fig. 4D). Fae7262 showed the highest yield at pH 7.0 (35.7%) while Fae68 at pH 6.0 (33.2%). When L-arabinose was used as acceptor, highest yields and rates were observed in the range of pH 6–8 (25.1–29.0% and 0.078–0.090 mol AFA g^−1^ FAE L^−1^ h^−1^) while the highest selectivity was observed at pH 8.0 (0.807). AFA synthesis catalyzed by Fae7262 and Fae68 was negligible.

### 3.8 Effect of Temperature

**Fig. 5:**
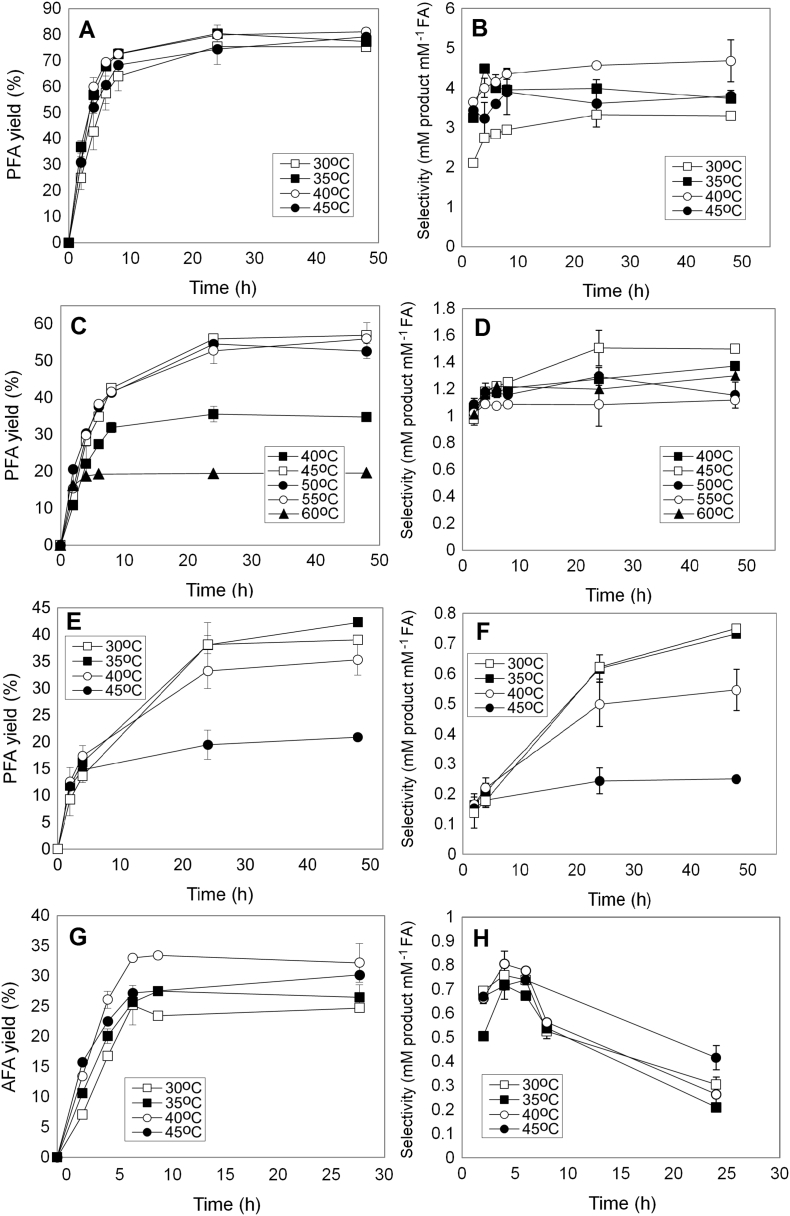
Effect of temperature on the yield and selectivity. Reactions were carried out at optimal medium composition, substrate concentration, enzyme load and pH for each enzyme. A, B, G, H): Fae125; C, D): Fae7262; E, F) Fae68.

Transesterification was monitored at different temperatures (25-60 °C) with respect to time at optimum conditions for each enzyme. Among the tested FAEs, Fae125 exhibited the highest PFA yield (81.1%) after 24 h of incubation at 35-40 °C followed by Fae7262 that exhibited 56% yield after 24 h at 45-55 °C (Fig. 5A and C). For both enzymes, the selectivity was increased during the first 8 h of incubation and stabilized thereof (Fig. 5B and D) while it was affected by the operating temperature. Highest selectivity was observed at 40 °C (4.685) for Fae125 and at 45 °C for Fae7262 (1.572). Fae68 showed lower rates achieving a 42.3% PFA yield after 48 h of incubation at 35 °C (Fig. 5E). Selectivity increased over the 48 h of incubation reaching a value of 0.733 at 30 or 35 °C (Fig. 5F). Regarding AFA synthesis, only Fae125 showed increased transesterification potential yielding 33% after only 8 h of incubation at 40 °C (Fig. 5G). The selectivity increased during the first 4 h of incubation at a range of 30-40 °C while at 45 °C the increase took up to 6 h decreasing up to 70% until the 24th hour (Fig. 5H). The hydrolysis of AFA after a critical ratio of VFA and AFA has been reached is strongly connected with the increased catalytic efficiency of Fae125 and by the composition of the reaction system, as L-arabinose is introduced as part of the water phase being in direct contact with the enzyme. A summary of the optimal conditions achieved in this work is presented in Table 3.

**Table 3:** Summary of optimal conditions and obtained parameters for PFA synthesis.

Bioconversion	PFA synthesis			AFA synthesis^a^
Enzyme	Fae125	Fae7262	Fae68	Fae125
Optimized conditions				
Water content (% v/v)	3.2	3.2	5.0	5.0
System composition	System II	System II	System I	System I
VFA concentration (mM)	60	100	60	80
Prenol or L-arabinoseconcentration (M)	1.5	1.5	0.8	0.5
Enzyme concentration (g FAE L^−1^)	0.04	0.05	0.05	0.02
pH	6-8	7	6	6
Temperature (°C)	40	45	35	40
Time (h)	24	24	48	6

Obtained parameters				
Yield (%)	81.1 ± 1.4	56.0 **±** 2.1	42.3 **±** 0.2	33.0 **±** 0.6
Overall VFA conversion (%)	98.5 **±** 0.3	93.3 **±** 0.2	100 **±** 0.0	75.5 **±** 5.2
PFA or AFA concentration (mM)	48.7 **±** 0.8	56.0 **±** 2.1	25.4 **±** 0.1	16.5 **±** 0.3
Rate (mol product g^−1^ FAE L^−1^ h^−1^)	0.051 **±** 0.001	0.050 **±** 0.002	0.011 **±** 0.000	0.138 **±** 0.003
Initial rate (mol product g^−1^ FAE L^−1^ h^−1^)	0.213 **±** 0.006	0.141 **±** 0.003	0.049 **±** 0.006	0.326 **±** 0.005
Selectivity (mM product mM^−1^ FA)	4.685 **±** 0.529	1.572 **±** 0.422	0.733 **±** 0.004	0.778 **±** 0.040

The errors represent the standard deviation between duplicate experiments.

a AFA synthesis by Fae7262 and Fae68 was negligible (<5%) after optimization of reaction conditions.

The optimization of reaction conditions using three FAEs belonging to different phylogenetic subfamilies revealed that the acetyl xylan related FAEs Fae125 (Type A, SF5) and Fae7262 (Type B, SF6) had higher synthetic potential than the tannase related Fae68 (Type B, SF1). To the authors' knowledge, the achieved PFA yield (81.8%) by Fae125 is the highest reported for the transesterification of a lipophilic feruloyl derivative catalyzed by a FAE in free-form to date. Use of immobilized FAEs for the transesterification of methyl hydroxycinnamates with 1-butanol has resulted into higher yields (78–97%) in certain cases with the exception that the acceptor was utilized as component of the reaction medium [[20], [21], [22]]. Moreover, the obtained selectivity (4.685) was 2-fold higher than the one achieved by free-form FaeB2 from *M. thermophila* (2.373) for the synthesis of PFA in detergentless microemulsions [11]. This can be linked with the increased stability of Fae125 in high prenol concentration (1.5–3.0 M) favoring transesterification over hydrolysis (Fig. 3C). This was not observed in the case of FaeB2 since the transesterification rate decreased by 60% with an increase of prenol concentration from 1 M to 3 M [11]. The achieved AFA yield (33%) by Fae125 in this work is comparable with the one achieved by FaeA1 from *M. thermophila* C1 in a similar study (52.2%) [12]. However, Fae125 operates optimally at lower temperature (30-45 °C), wider range and reduced incubation time (6 h for AFA) compared to FaeA1 (45-55 °C, 8 h), despite the weaker performance in AFA synthesis. A great advantage of Fae125 as biocatalyst is its ability to transesterify VFA with small/lipophilic and bulkier/hydrophilic acceptors such as prenol and L-arabinose, respectively, at competitive yields utilizing a single enzyme. Thus, Fae125 could prove an attractive biocatalyst for application in the modification of hydroxycinnamates due to its ability to operate at high acceptor concentrations without significant deactivation and low temperatures.

### 3.9 Effect of Donor and Agitation

**Fig. 6:**
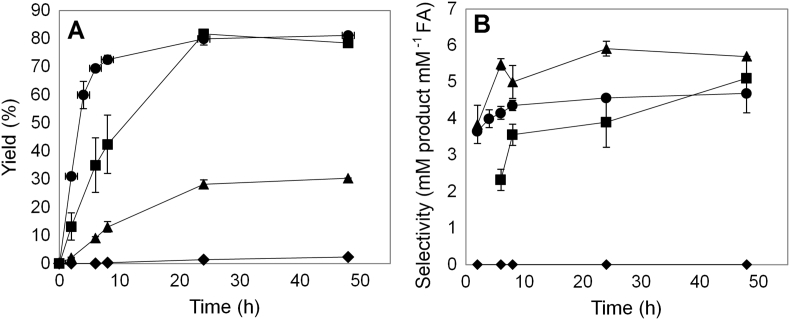
Effect of other donors and agitation on the A) yield and B) selectivity using Fae125. Circle: VFA, no agitation, Square: VFA, 1000 rpm, Triangle: MFA, 1000 rpm, Rhombus: FA, 1000 rpm.

The possibility of substituting VFA with other commercial donors (MFA or FA) and the effect of agitation were investigated at optimized conditions offering the highest yield (PFA synthesis by Fae125). As expected, VFA was a more reactive donor while transesterification with MFA resulted in 30% PFA yield after 48 h of incubation (Fig. 6A). Direct esterification was negligible. Agitation (1000 rpm) impacted the initial rate resulting in 50% decrease; however, at 24 h the yield was similar for reaction with and without agitation. The same trend was observed for the selectivity which increased over 4.0 after 25 h of incubation (Fig. 6B).

### 3.10 Comparison of Fae125 with FaeA1 from *M. thermophila* C1

**Fig. 7:**
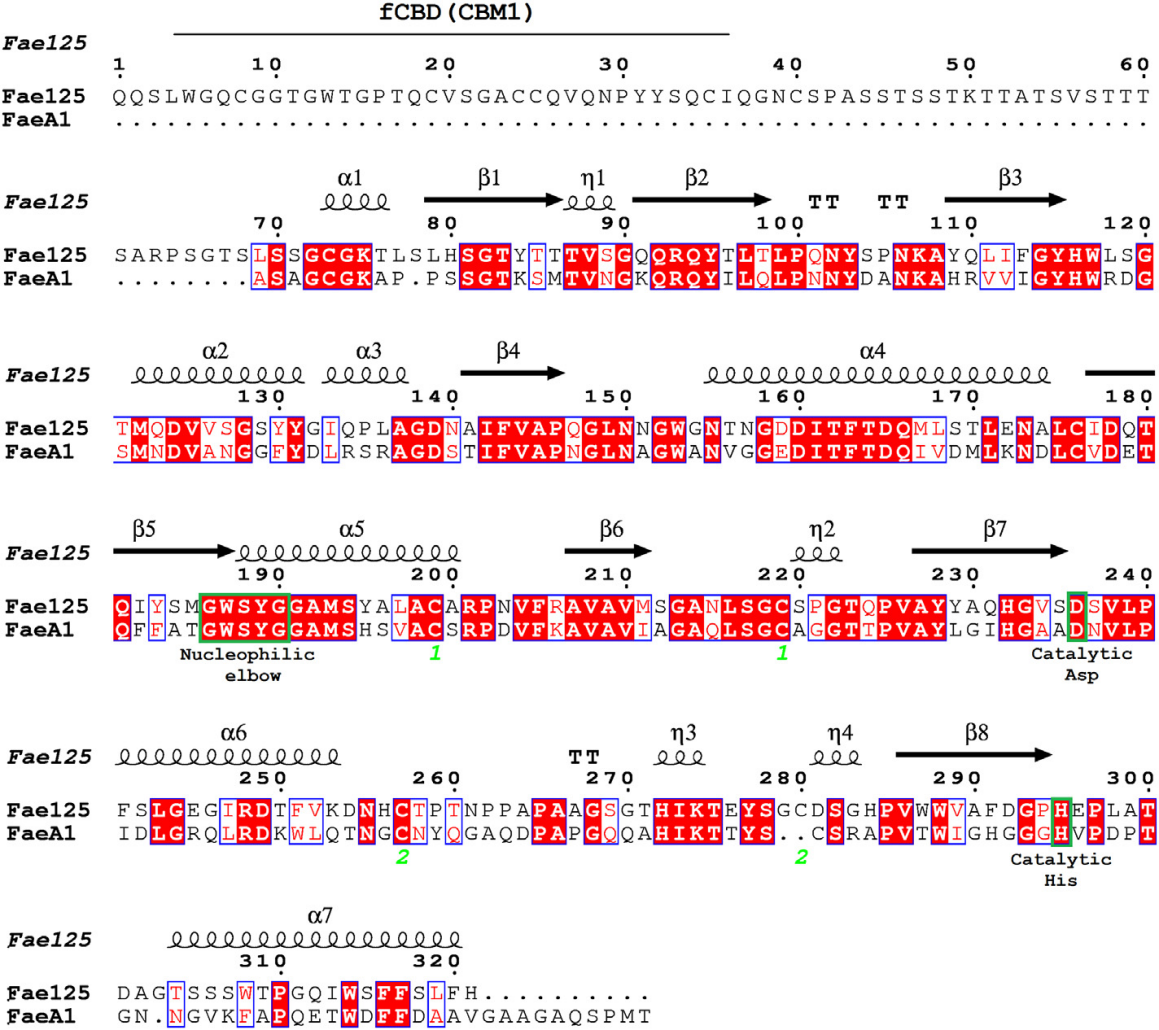
Sequence alignment by ESPript of Fae125 and FaeA1. The predicted secondary structure elements of Fae125 are presented at the top of the alignment (α-Helices, 3_10_-helices, β-sheets and strict β-turns are denoted α*,* η*,* β and ΤΤ, respectively. Τhe numbers 1 and 2 indicate the position of disulfidic bonds).

Fae125 is an enzyme very similar to FaeA1 from *M. thermophila* C1 in terms of primary sequence (51% identity; 67% similarity; 1% gaps; 2·10^9^ *E*-value) (Fig. 7). However, the poor homology of SF5 acetyl-xylan related enzymes to known structures poses severe limitations to the comparison of enzymes such as Fae125 and FaeA1 regarding their difference in transesterification activity. In this work, the prediction of the secondary structure of Fae125 was done by homology modeling in YASARA Structure based on five template PDB entries (1JJF, 1JT2, 3WYD, 3PE6, 4BMF). Twenty-one models were built and assorted by their overall quality scores (*Z*-score) and the final model was a hybrid combining the best parts of produced models (residues 53–321, 1JJF; residues 132–137 and 236–247, 3WYD; residues 272–275, 3PE6). The hybrid model resulted in good quality scores regarding the secondary structure (−0.410 for dihedrals and − 0.9501 for 1D packing). As expected and due to no significant homology to known structures, the quality of 3D packing of Fae125 was poor (−2.589) and, therefore, conclusions cannot be drawn on the activity or the binding cavity formation of the enzyme. The hybrid model had a satisfactory overall quality score (−1.634). The catalytic triad of Fae125 is predicted as: Ser188-Asp236-His295 and a cellulose binding domain (CBM1) is predicted at residues 4–30. The secondary structure of Fae125 is presented in Fig. 7. Looking into particular motifs on the primary sequences of the two enzymes, it can be seen that there is a difference in the hydrophobicity around the residues of catalytic triad, although the enzymes have similar hydrophobicity as molecules within the aligned region (Fae125 39%; FaeA1 41%). The hydrophobicity of the two enzymes around the nucleophilic elbow is identical. However, Fae125 is approximately 20% more hydrophobic than FaeA1 around the catalytic His residue (residues 290–303). This could explain the good performance of Fae125 with prenol and the poor performance with L-arabinose, as lipophilic acceptor molecules could be more easily attracted near the active site and enable transesterification. Nevertheless, research should focus on the structural determination of acetyl xylan-related FAEs, in order to associate the structural properties of the subfamilies 5 and 6 with their synthetic performance and the preference in different acceptors.

## 4 Conclusions

In this work, we evaluated the synthetic potential of three FAEs from *T. wortmannii* in detergentless microemulsions by optimization of reaction conditions aiming to the synthesis of two compounds of different lipophilicity: PFA and AFA. The type A Fae125, an acetyl-xylan related enzyme belonging to the SF5 phylogenetic subfamily, was proved potent biocatalyst being able to transesterify VFA with both lipophilic prenol and hydrophilic L-arabinose in competitive yields. The acceptor and enzyme concentration were identified as the parameters with highest impact on the selectivity. After optimization, significant increase in the yield (8-fold) and selectivity (12-fold) were achieved for the synthesis of PFA aiding into the development of robust FAE-based biocatalytic processes for bioactive compound modification.

## Author Contributions

IA (Luleå University of Technology, Sweden) designed and performed the experiments, analyzed the data and wrote the manuscript; UR and PC designed the experiments and analyzed the data; LI (DuPont Industrial Biosciences, the Netherlands) provided the enzymatic preparations Fae68, Fae7262 and Fae125; PJ (Taros Chemicals, Germany) provided VFA as donor for enzymatic transesterification; All authors reviewed and approved the manuscript.

## Declaration of Interest

None.
